# Cocaine

**Published:** 1894-07

**Authors:** Arthur R. Cushny


### Cocaine.

BY ARTHUR R. CUSHNY, A.M.,M.D.

Read before the 38th Annual Meeting of the Michigan State Dental Association.

Cocaine was first introduced into medical practice in 1884 although its action had been described some 18 years before. Between 1884 and 1889 about 114 cases of poisoning occurred of which 6 proved fatal. The publication of these figures has resulted in deterring many surgeons and others from the use of a most useful drug. On examination, however, we find that in many of these cases what must be characterized as enormous doses were used, for in two cases which I have looked up the doses used were 12 and 20 grains respectively. Cocaine belongs to the group of the alkaloids, and these, as you know, comprise some of the most powerful poisons. Very few of them could be injected in doses of 5 grains, and cocaine is no exception to the rule. To discard all drugs that are poisonous in doses of 12–20 grains would result in removing from the pharmacopia almost all the most useful active principles.

Cocaine acts principally on the peripheral endings of the sensory nerves and on the central nervous system. The terminations of the sensory nerves are completely paralyzed, causing anæsthesia of the part to which cocaine has been applied. This paralysis is however not confined to the peripheral endings, for recent investigation has shown that sensory nerve trunks may also be paralyzed by cocaine, that is, the conducting power of nerves is destroyed. Whether it would be advisable to use this paralysis of the nerve trunk for anæsthesia is, I think, open to question, for such interference with the trunk of a nerve is liable to set up neuritis.

The peripheral action of cocaine is that which is made use of chiefly in practice. Its action on the central nervous system is only seen in cases of poisoning, and in rare cases where it is used internally as a stimulant. Schroff described this central action many years ago, and it has been in use in South America since time immemorial for its action on the brain. Anrep studied its action on the dog and described it as a powerful stimulant to the brain. The dog immediately after the injection becomes excited, and cannot stand still but runs about, leaping and dancing on his hind legs without cessation. The movements are altogether different from those seen in strychnine tetanus. There is no convulsive movement, no tonic contraction. The action seems to be on the highest spheres of the brain. The movements are those of a pet dog meeting his long-lost master. This stage lasts one to three hours and then gradually passes off, the animal becoming normal again.

On larger doses being given the stage of excitement is still greater, but then one sees distinctly that the dog has no longer control over himself—he runs in a circle, and when called evidently tries to approach, but cannot leave off the constant movement, in fact, his motor centers have run away with the animal. If caught he is found to be restless, scarcely recognizes his owner; is very easily frightened and trembles violently. After some time this stage passes off, and languor and exhaustion set in, but he recovers in a few hours completely. On giving 1-3 grain per kilo a short stage of excitement is seen but very soon great exhaustion follows, then convulsions, unconsciousness, stupor. The explanation of these symptoms is extreme stimulation of the cortex. This stimulation is followed by weakness and paralysis of the centers. In man this stimulation is also seen often in smaller doses—talkativeness, disposition to movement, laughter, general jollity. More marked, however, in man is the stage of paralysis of the brain shown by giddiness, langour, feeling of weakness and faintness, pallor, indistinct vision, unconsciousness which may go into collapse. The pupils are widened by stimulation of the sympathetic terminations in the iris. The respiratory center is first stimulated. A curious feature is a sort of Cheyne-Stokes respiration which appears in severe poisoning. Even with small quantities it is found that the breath can be held much longer than normally. The respiratory standstill is the cause of death. The heart itself is comparatively unaffected, but the vagus is paralyzed and it is therefore quickened. In very large doses it may be weakened and slowed by direct action of the drug on the heart muscle. All the mucous and salivary secretory glands are weakened, and patient complains often of dryness of the throat and tongue.

In conclusion, I think I am justified in saying that the disrepute into which cocaine has fallen in a section of the profession is undeserved. The smallest dose which has hitherto been shown to produce any dangerous symptoms is 1 1-4 grains, and this is more than is sufficient for any of the smaller operations and is far more than is required for, at any rate, one tooth. The only point to be remembered, is that the cocaine must be pure, and that it ought to be used in about four to five per cent. solution. When impure cocaine is used, the blame should be borne by the manufacturer and operator, and should not be laid on the drug.

I should like to say a few words regarding the chemical nature of cocaine, as it is probable that before many years are over several new allied preparations will be put on the market. Cocaine like so many of the vegetable alkaloids is a derivative of pyridine. In it the pyridine ring is combined with oxypropionic acid to form ecgonine. The ecgonine is then combined with methyl and the hydroxyl group substituted by benzoyl. In coca we find several other alkaloids closely resembling cocaine in their composition and action, such as cinnamyl cocaine in which the hydroxyl group is substituted by cinnamyl instead of benzoyl and the truxillines in which the molecule is doubled. Besides these, several cocaines have been formed artificially and we seem to be approaching the time when we will no longer depend on the coca leaf for our supply of cocaines because they will be manufactured synthetically. Moreover, the molecule of cocaine can be altered so greatly and still preserve its anæsthetic properties, that I think we may very soon form a base which possesses all the virtues and none of the disadvantages of cocaine.

Ecgonine is very nearly related to the tropin radicle found in atropine, hyoscine, etc., and a new alkaloid has been described lately which belongs to the tropeins or rather the pseudotropeins, but possesses the cocaine anæsthetic action. This new alkaloid is Tropacocaine. As you are aware atropin and the allied bodies have been long known to possess slight local anæsthetic powers. This power is much increased however in tropacocaine, which, as a local anæsthetic, promises to prove a formidable rival to cocaine. Its pharmacological action has been studied by Chadbourne, who attributes to it a considerably greater anæsthetic action than cocaine with less pronounced toxic properties. Its anæsthetic properties have been testified to by several ophthalmic surgeons, but its use has been too limited as yet to admit of my discussing whether its toxic action is greater or less than that of cocaine. Here again, however, we have another series of bodies possible which may prove to be of great service as local anæsthetics, and, if the pseudotropeins prove to be less dangerous than the cocaines, we may possibly find in a few years cocaine dropping out of use and its place being taken by a synthetically manufactured pseudotropein.

